# Does Breast Milk Nurture T Lymphocytes in Their Cradle?

**DOI:** 10.3389/fped.2018.00268

**Published:** 2018-09-27

**Authors:** Peter S. Hsu, Ralph Nanan

**Affiliations:** ^1^Allergy and Immunology, Kids Research, The Children's Hospital at Westmead, Sydney, NSW, Australia; ^2^Discipline of Child and Adolescent Health, The University of Sydney, Sydney, NSW, Australia; ^3^Charles Perkins Centre Nepean, University of Sydney, Sydney, NSW, Australia

**Keywords:** breast feeding, thymus, immunity, breast milk microbiome, regulatory T cells

## Abstract

Breast feeding has been associated with improved infant outcomes in multiple aspects, including immune outcomes such as infections and potentially atopy and autoimmunity. However associations do not necessarily implicate cause and effect and at this point, exactly how breast feeding and components of breast milk may modulate the infant's immune compartment remains unclear, especially in humans. Some lines of evidence suggest that breastfeeding affects the development of the infant's thymus, a critical organ for T cell development. This may be a direct effect mediated by breast milk components or alternatively, a secondary effect from the impact of breast feeding on the infant's gut microbiome. Here we discuss the potential mechanisms and impact of this association between breast feeding and thymic development.

## Introduction

Thymus derived lymphocytes are one of the central players of the adaptive immune system. Adaptive immune responses in mammals are characterized by extreme specificity in terms of antigen recognition. Such highly tailored responses have evolved as a powerful defense strategy against rapidly changing pathogens resulting in improved host survival. A broad and effective development of adaptive immune responses is eminently important in the newborn period, when the offspring is exposed to a range of environmental pathogens it shares with its mother. Here mammals, in contrast to reptiles, are provided with the unique opportunity to shape thymic and T cell development in the offsprings for a prolonged period after birth by means of lactational immune programming.

Human breast milk contains a wealth of bioactive substances including proteins, oligosaccharides, polyunsaturated fatty acids, micronutrients, metabolites and microbial components. Many of these bioactive constituents can modulate the infant immune system in the critical first year of life. Whilst the protective effects of maternal IgA in breast milk for neonatal infections is well established (1), the role of the many other immunologically active components in breast milk is less clear. Notably despite the heterogeneity of various studies and the lack of randomized controlled trials, there is evidence that breastfeeding may influence long-term infant immune outcomes such as allergic and gastrointestinal diseases (2). Here we review the current evidence for the influence of breastfeeding on the infant immune system, with a focus on the thymus, a critical organ for T cell immune development.

## Breast feeding and infant immune outcomes

In addition to *in utero* immune programming, breast feeding offers an early postnatal route by which the mother may pass on protective factors to her infant and shape the developing immune system. Perhaps the best established data for the effect of breastfeeding on infant immunity is its protective effect for neonatal respiratory and gastrointestinal infections. It is thought that breast milk immunoglobulin A (IgA) secreted by activated resident switched memory B cells in mammary glands, is the main proponent for this effect, as it supplements the low neonatal IgA production (3). Additionally, secretory IgA from breast milk is also thought to promote infant immune tolerance to various antigens by reinforcing the gut epithelial barrier and reducing pro-inflammatory responses (4).

In addition to this short term protection, breast feeding may be associated with improved infant immune outcomes. These studies consist of observational and cohort studies and due to variation in study methodology and definitions, the results are more mixed. One prospective cohort study found no significant evidence that infant feeding practices in the first 6 months influenced the development of wheeze, asthma or atopic dermatitis (5). Another study found that increased duration of breast feeding was associated with protection against non-atopic asthma (6). A meta-analysis including studies up to 2014 suggested that breast feeding was protective for asthma and children who were exclusively breastfed for more than 3–4 months have a lower risk of developing eczema under 2 years of age, however potential recall bias and lack of confounder adjustments in some of the included studies may have influenced the analysis (7). Some studies also show that breast feeding may protect against food allergy (8, 9), however other studies suggest otherwise (10, 11). In terms of autoimmune disease, breast feeding appears to confer protection to the infant for both type I and II diabetes (12) and multiple sclerosis (13). However evidence for a protective effect on other autoimmune diseases such as arthritis and inflammatory bowel disease is controversial (14).

Several putative mechanisms have been put forward regarding the reason for the potential immune protective effect of breastfeeding. Reduced neonatal infections in breastfed infants is thought to contribute to the reduced rate of asthma in these children, as early and recurrent respiratory infections have been linked with development of asthma (15). In addition to IgA, there are a multitude of immune active substances including immune cells, cytokines and microbiome in the breast milk, which may be involved in infant immune programming (2). However due to the significant variability in breast milk components (16), it has been difficult to study or isolate the effect of these substances in humans. Mechanistic studies in mice showed that presence of allergen (via maternal exposure to allergen during lactation) with TGFβ, or IgG/antigen immune complex in breast milk led to protection against allergic asthma in the progeny via induction of antigen specific Treg cells (17, 18). This is supported by a systemic review of human studies, which revealed that two thirds of the studies showed an association between increased TGFβ levels in breast milk with reduced atopic outcomes (19). Soluble CD14 (sCD14), a co-receptor for bacterial cell wall components such as lipopolysaccharides (LPS) is also found in variable concentrations in human breast milk (20). High breast milk sCD14 level has been associated with protection against atopic disease (21), possibly by enhancing Th1 immune responses, though the exact immune mechanism is unclear.

Another significant influence of breast milk on the infant is the presence of breast milk microbiota and the effect of its colonization on the infant immune system. Indeed, the human breast milk contains a variety of viable bacteria (22) and there are early significant differences in the gut microbiota between exclusively breastfed and formula-fed infants (23), suggesting the gut microbiome may affect allergy development. However exactly how the breast milk microbiota may influence infant gut microbiome and immune outcome needs to be interrogated in future studies.

Many other immune active substances have been found in the breast milk including human milk oligosaccharides (HMO), which are thought to be protective against mucosal infections (24) and may be linked to reduced development of allergy in the infant (25).

## The thymus

The thymus is a critical center for T cell development. Developmentally, it is derived from the pharyngeal arches and is formed and functional at around 14–16 weeks of gestation (26). Lymphoid progenitors migrate from the bone marrow to the thymus, where they undergo a series of differentiation checkpoints. Briefly, the important processes include the development of formation and rearrangement of T cell receptor, acquisition of important surface markers such as CD3, CD4, CD8, and positive selection (where cells that bind inadequately to self MHC/antigen complex are deleted), then finally negative selection (where cells binding too strongly to MHC/self-antigens are deleted) (27). This results in the continual generation and emigration of naïve T cells from the thymus. Foxp3+ regulatory T cells (Tregs), a critical immune cell subset for immune tolerance, are also generated in the thymus in a similar manner, except their development requires a differential strength of signaling from self-antigen/MHC complexes, so that they are favored to suppress unwanted immune responses to self-antigens (28). The importance of the thymus is seen in cases of complete failure of thymic development (thymic aplasia) such as in of 22q11.2 deletion and CHARGE syndrome, which leads to severe combined immune deficiency with absent T cells and poor T cell dependent antibody responses (29).

Despite its important role in T cell generation, in all vertebrates the thymus involutes with age. Thymic stromal cells and architecture begin to regress as early as the first year of life, with disorganization of cortico-medullary junction and replacement of thymic tissue with adipocytes (30). The rate of regression increases to about 3% per year during adulthood (31). This thymic involution is thought to be an important contributor to immunosenescence, which results in reduced naive T cell output, restricted T cell receptor repertoire, impaired responses to pathogens and vaccines, thereby leading to increased susceptibility to infections, autoimmune diseases and malignancy (32). A similar process is brought forward in infants who have early thymectomy for cardiac surgery, who show impaired thymic output with reduced T cell numbers and function, as well as immunoglobulin levels (33, 34). Several studies have attempted to examine the impact of thymectomy on clinical outcomes (35, 36). Whilst no significant morbidities were noted in the thymectomized population, it is important to note that thymic regrowth occurs in the majority of cases (37), making it difficult to assess the true impact of postnatal absence in thymic tissue. Furthermore the timing of thymectomy is quite variable, which may also influence the magnitude of the clinical impact.

## A link between breastfeeding and thymic development

A study by Hasselbalch et al. more than 20 years ago found that there was a correlation between breast feeding and thymic size (38). In a small cohort of 47 healthy infants, they assessed the thymic size at birth and 4 months of age using trans-sternal ultrasound measurement. The geometric thymic index was derived from the multiplication of the largest transverse and sagittal diameter on ultrasound. They found that the thymic index was significantly higher in babies who were exclusively breastfed compared to partially breastfed (infants who have breast milk at least once a day) and exclusively formula fed infants. However there was significant overlap between the groups. The authors concluded that the thymus is significantly larger breast-fed infants. A follow-up study from the same group recruited a different cohort of 50 partially breastfed infants at 8 months (39). The thymic index was derived by the same methodology. In this study they found that at 10 months of age, those infants who had continued to have some breast milk at 10 months had a slightly larger thymus compared to those who have stopped breast feeding. However the difference was very small with significant overlap. Notably these infants were all breast fed for at least 8 months, and therefore one might not expect a significant difference with extra 2 months of breast feeding. Importantly, both of these studies used trans-sternal ultrasound to assess the thymus size postnatally, which may be problematic due to echogenic shadow from the overlying bone. Additionally, how well thymic size correlates with thymic function and output is unclear and neither of these studies specifically examined thymic output.

In a subsequent paper, Jeppesen et al. examined the CD4 and CD8 counts and proportion in infants between 1 and 12 months of age (40), where they found some evidence of reduced CD8 and CD4 percentages in infants who had stopped breast feeding at 8 or 4 months of age respectively. They also found a modest correlation between the frequency of breast feeding and CD4 T cell counts, but not between thymic size and T cell counts. This study provides some evidence that breast feeding may influence the T cell compartment in infants, but fails to show that this influence is mediated through the thymus. Indeed other measures of thymic output or function would be needed to clarify this issue. A good candidate would be measurement of T cell receptor excision circles (TREC), which are genetic by products of T cell receptor recombination. TREC level is higher in naïve T cells and becomes diluted with T cell activation and division, therefore it serves as a good marker for thymic output of naïve T cells (41). Alternatively, measurement of recent thymic emigrants by their expression of CD45RA and CD31 with flow cytometry could also be used to monitor thymic output.

Additionally, in looking at immune outcomes in breast fed infants, one should also examine the effect of breast feeding on thymic output of Treg cells, since they are integral for controlling allergic and autoimmune responses. Unfortunately at this stage no study has examined this aspect in humans.

## Breast milk components that may affect thymic development

Several components in breast milk can potentially modulate the T cell mediated immunity by directly or indirectly shaping thymic development. An interesting study conducted in rural Gambia showed that infants born in the “hungry season” had smaller thymuses, associated with significantly higher adult mortality due to infectious diseases, compared to those born in harvest season (42). A later study showed that the reduced infant thymic size in the hungry season was associated with reduced thymic output as measured by TRECs. Furthermore, they showed that breast milk of mothers in the hungry season had significantly lower interleukin 7 (IL-7) levels compared to harvest season (43). These findings are correlative, but since animal studies have shown that breast milk cytokines can be passively transferred to the infant's systemic circulation (44), it does suggest that breast milk IL-7, an important factor for T cell thymopoeisis (45), may play a role in infant thymic development. Nevertheless, many other breast milk components may potentially differ in hungry and harvest season to influence infant thymic development.

Another interesting component in breast milk is the highly conserved RNA packaged in exosomal particles, known as microRNA (miR). These miRs are able to regulate immune cell development at the post-transcriptional level (46). In particular, murine models show that miR-155 plays an important role in regulating thymic Treg cell and Th2 cell development (47). Another miR, miR-449a appears to be important for regulating thymic medullary epithelial cell development in mice (48). Importantly, human breast milk contains abundant miRs (including miR-155) which may regulate infant immunity (49). Hence, transfer of miRs might be implicated in regulating infant thymic development and immunity.

In addition to bioactive molecules, data from a murine model indicates that viable maternal CD4+ immune cells are transmitted through breastmilk and home to the offspring's thymus. Here they appear to play an important role in educating the CD8+ T cell compartment. This might be an important mechanism to prepare the offspring against pathogens like mycobacteria or other persistent intracellular pathogens, which the offspring might encounter in the shared maternal environment (50). Another murine study showed that maternal cytotoxic T lymphocytes in breast milk are able to localize to the Peyer's patch of the nursed infant (51). Overall it is conceivable that maternal immune cells, especially T cells, may be passively transferred to the infants via breast milk. There is some evidence at least in the animal model that these maternal T cells may traffic to the infant thymus, where they may educate and influence infant T cell repertoire, thereby potentially affect infant immune outcomes.

Hormonal levels in human breastmilk may also have an important potential to influence thymic development. For example, corticosteroids are known to regulate thymic epithelial differentiation and the formation of Hassel bodies (52). Cortisol level varies significantly in breastmilk depending on the stress levels of the mother and the circadian rhythm (53), therefore these factors may also influence the infant thymic development. In addition, the actual act of breastfeeding and nursing might also have a soothing effect on the suckling child and hence influence the infants own corticosteroid output impacting thymic function. Indirect evidence for this was provided in a mouse model where hand- but not brush-stroked mice demonstrated a significant increase in thymic and splenic T cell number (54).

## Effects of breast milk on the gut microbiome

Human breast milk itself contains a significant bacterial load which influences the colonization and development of the infant gut microbiome (55). In addition, human milk oligosaccharides (HMO) may help to establish a healthy gut microbiome in the infant by regulating the gut epithelium, immune cells and micro-organisms (56). Among the many metabolites produced by the gut microbiome, short chain fatty acids (SCFA) have been shown to exhibit powerful immunomodulatory effects especially on T cells. Most recently it has been shown that high fiber diet during pregnancy and lactation fuels the production of SCFA in mice, which may act directly via SCFA receptor in the thymic microenvironment to enhance thymic output of Treg cells (57). Therefore breast milk may potentially modulate infant thymic development by influencing infant microbiome composition and bacterial metabolite production.

## Summary and future directions

**Figure 1:**
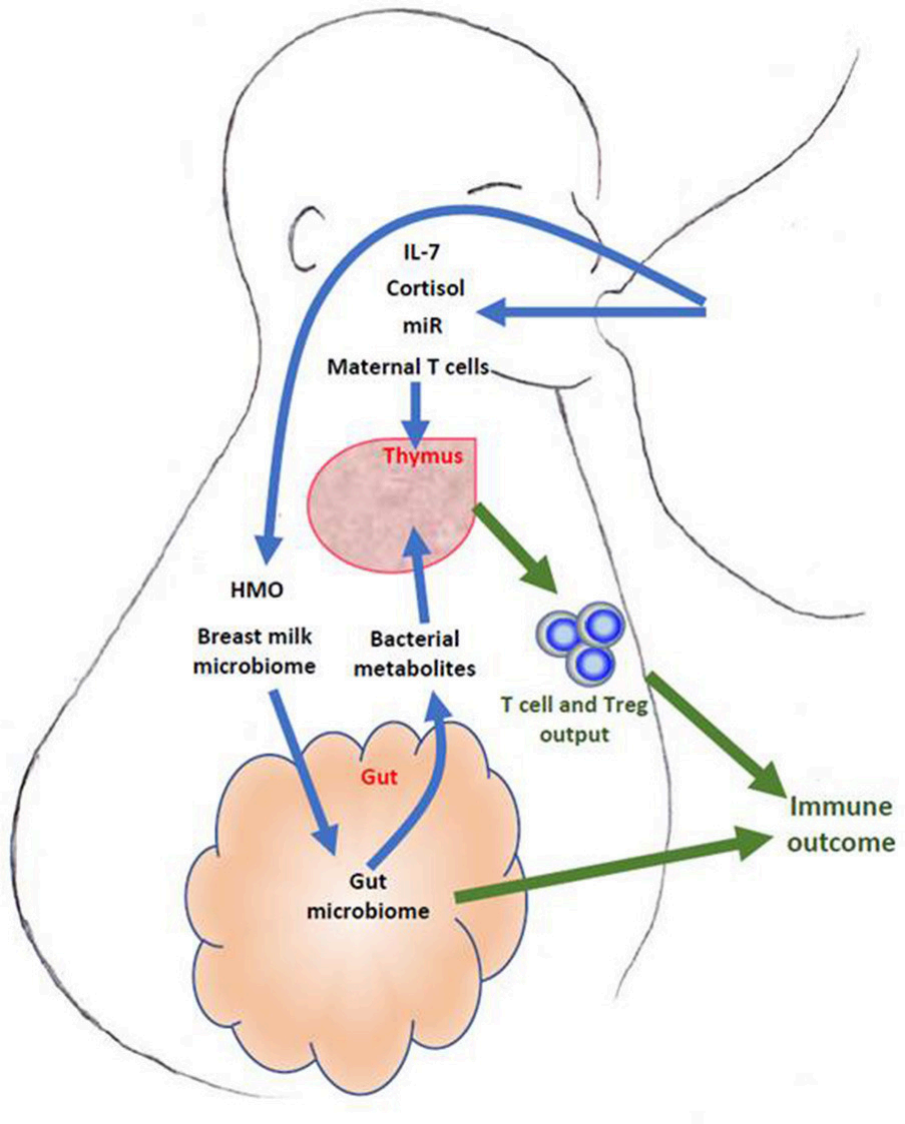
Schematic description of breast milk components that may influence fetal thymic development and output. Some such as IL-7, micro-RNA (miR), hormones and maternal T cells may act directly through the thymus, whilst others such as breast milk microbiome, human milk oligosaccharides (HMO) may act via the gut microbiome and its metabolites.

Overall, there is significant evidence from animal models that breast feeding may influence infant thymus and hence T cell development. However there are currently no data regarding whether such thymic changes may influence long term immune outcomes. Further investigations into the possible influence of maternal breast milk on human thymic development are required. Many bioactive components of the breast milk including cytokines, hormones, micro-RNAs, oligosaccharides, as well as active maternal immune cells and mammary microbiome, may modulate the infant immune development through mechanisms both dependent and independent of the thymus. The main concepts are summarized in Figure 1. However most of the mechanistic studies have been performed in animal models, where milk components and their effects may be very different. Furthermore, there is a lack of direct evidence linking the effect of breast milk components to thymic output and infant immune outcomes. This is partially due to the lack of specific and systemic approaches in the measurement of thymic output and breast milk components in parallel with long term immune outcome follow-ups. Future well-designed longitudinal studies would be required to address these issues. These studies may enable us to design strategies to modulate lactational immune programming for the primary prevention of immunopathologies such as autoimmune disease and allergies.

## Author contributions

All authors listed have made a substantial, direct and intellectual contribution to the work, and approved it for publication.

### Conflict of interest statement

The authors declare that the research was conducted in the absence of any commercial or financial relationships that could be construed as a potential conflict of interest.
